# Low Metal Loading (Au, Ag, Pt, Pd) Photo-Catalysts Supported on TiO_2_ for Renewable Processes

**DOI:** 10.3390/ma15082915

**Published:** 2022-04-15

**Authors:** Francesco Conte, Ilenia Rossetti, Gianguido Ramis, Cyril Vaulot, Samar Hajjar-Garreau, Simona Bennici

**Affiliations:** 1 Chemical Plants and Industrial Chemistry Group, INSTM Unit Milano-Università, Dip. Chimica, CNR-ISTM, Università degli Studi di Milano, via C. Golgi 19, 20133 Milan, Italy; francesco.conte@unimi.it; 2 INSTM Unit Genova, Dip. Ing. Chimica, Civile ed Ambientale, Università degli Studi di Genova, Via all’Opera Pia 15A, 16145 Genoa, Italy; gianguidoramis@unige.it; 3 Institut de Science des Materiaux, CNRS, IS2M UMR 7361, Université de Haute-Alsace, F-68100 Mulhouse, France; cyril.vaulot@uha.fr (C.V.); samar.hajjar@uha.fr (S.H.-G.)

**Keywords:** photo-catalysis, TiO_2_, CO_2_ photo-reduction, photo-reforming, photo-reactor

## Abstract

Photo-catalysts based on titanium dioxide, and modified with highly dispersed metallic nanoparticles of Au, Ag, Pd and Pt, either mono- or bi-metallic, have been analyzed by multiple characterization techniques, including XRD, XPS, SEM, EDX, UV-Vis and N_2_ adsorption/desorption. Mono-metallic photo-catalysts were prepared by wet impregnation, while bi-metallic photocatalysts were obtained via deposition-precipitation (DP). The relationship between the physico-chemical properties and the catalyst’s behavior for various photo-synthetic processes, such as carbon dioxide photo-reduction to liquid products and glucose photo-reforming to hydrogen have been investigated. Among the tested materials, the catalysts containing platinum alone (i.e., 0.1 mol% Pt/TiO_2_) or bi-metallic gold-containing materials (e.g., 1 wt% (Au_x_Ag_y_)/TiO_2_ and 1 wt% (Au_x_Pt_z_)/TiO_2_) showed the highest activity, presenting the best results in terms of productivity and conversion for both applications. The textural, structural and morphological properties of the different samples being very similar, the main parameters to improve performance were function of the metal as electron sink, together with optoelectronic properties. The high activity in both applications was related to the low band gap, that allows harvesting more energy from a polychromatic light source with respect to the bare TiO_2_. Overall, high selectivity and productivity were achieved with respect to most literature data.

## 1. Introduction

Titanium dioxide is a multi-purpose material used in many commercial applications and it is recognized as a good candidate for the development of semiconductors for photocatalytic processes [1,2]. When it is used as a photocatalyst, the incident light causes the electrons to be promoted from the valence band (VB) to the conduction band (CB), leaving a hole (h^+^) in the lowest energy state [3]. For solar light application, it is extremely important to select materials that have a narrow band gap (i.e., difference between the energy level of VB and CB) in order to absorb light of longer wavelength, since the ultimate goal of photo-catalysis is to exploit the visible region of the spectrum [4]. The electron-hole couple can be used to perform redox reactions that involve the chemical species absorbed over the photo-catalyst surface [5]. The holes will oxidize the substrates when the active material is employed in processes such as biomass reforming, while photo-excited electrons are useful to perform endergonic reactions like CO_2_ and H_2_O photo-reduction [6,7]. Both the electron and the hole are used for the photo-reforming reaction, to reduce H^+^ to H_2_ and to oxidise organic pollutants contained in water simultaneously [8], whereas H_2_O oxidation or the consumption of a sacrificial hole scavenger (HS) are involved in CO**_2_** photoreduction.

Starting from the pioneering work of Fujishima and Honda [9], thousands of papers on the photo-catalytic activity of titanium dioxide and related compounds have been published so far [10,11]. Indeed, TiO_2_ based materials can be produced by low-cost synthesis and are resistant under most conditions, especially against photo-corrosion. The main drawbacks that limit the applicability of TiO_2_ at a larger scale are due to its wide band gap (BG) and fast electron-hole recombination. Both issues depend on the crystalline structure, since titanium dioxide exists in three polymorphs, which are anatase (BG = 3.20 eV), rutile (BG = 3.02 eV) and brookite (BG = 3.40 eV) [12,13]. Although such values are not the only ones widely accepted, the absorption of those semiconductors occurs in the UV-A region, allowing exploitation of less than 5% of the solar energy spectrum and severely limiting solar light application [14].

Various approaches have been developed in order to overcome this problem, such as thin layers preparation, doping and surface modification, e.g., through the dispersion of metallic nanoparticles over the photo-catalyst surface [15,16,17]. Among the possible co-catalysts, platinum is the most widely used metal because of its ability to develop a strong interaction with the TiO_2_ support [18], but also gold and silver are employed for enhancing the adsorption under visible light, due to the localized surface plasmonic resonance (LSPR) phenomenon [19,20,21]. Establishing a metal/TiO_2_ heterojunction modifies the charge transfer process on the semiconductor and determines the formation of a Schottky barrier [22]. The irradiation of the photo-catalyst is accompanied by electron flow from or to the metal isle (depending on work function value) and this decreases the recombination rate of the photogenerated electron-hole couple and enhances the charge separation [23]. Specifically, Pt is able to receive electrons from the titania surface and acts directly as reduction center for the adsorbed species, while Au injects electrons into the semiconductor [24]. In addition, combining metals over the same surface, such as Au/Ag and Au/Pt bi-metallic samples, is an effective way to further increase photo-catalyst activity [25]. The surface functionalization is usually carried out by photo-deposition, deposition-precipitation (DP) [21,26], wet impregnation (WI) [27], and by flame-spray pyrolysis (FSP) [28].

As for the selected applications, CO_2_ photo-reduction is particularly interesting because 40 billion tons of CO_2_ are released into the atmosphere each year. The objective is to convert the carbon dioxide into less harmful, or even useful, compounds, such as liquid fuels (e.g., organic acids, alcohols, etc.) [6,29]. TiO_2_-based materials have already proved to be effective in oxyanions photo-reduction in an aqueous environment [30] and also for CO_2_ activation, since the titania surface is able to interact with that molecule through the hydroxyl groups and oxygen vacancies [31,32]. The carbon dioxide is then reduced by subsequent injection of electrons to obtain, for instance, formic acid, methanol and methane [33,34,35].

On the other hand, the production of hydrogen implies the same type of mechanism, but occurs by the reduction of a proton or any other available oxidized hydrogen species. In addition, the focus is also on the reaction between the holes (photo-generated on the active material) and the organic compounds present in the reaction media; ideally, organic pollutants in water to accomplish simultaneous water treatment and hydrogen production [7,36]. Such organic compounds act as sacrificial HS, and, in general, alcohols or polyols, like glucose and other sugars, are reported to prevent holes’ accumulation in the valence band [21,27,37,38].

Although a similar mechanism is involved in both the photo-synthesis reactions (CO_2_ and H_2_O photoreduction), the similitude between the reactions and the features of the photocatalysts that are able to boost their activity and selectivity for both applications have not been fully clarified. Therefore, in this work we discuss the photo-catalytic activity of selected metal-promoted photo-catalysts for both these photo-synthetic applications, in light of the characterization results. Such extensive characterization has been missing so far and may lead to a better understanding of the photo-catalytic activity of our photo-catalysts. Specifically, the materials were made from commercial titanium dioxide (P25). The functionalization was carried out by DP and WI by depositing metallic Au, Ag, Pd, Pt, or various combinations of these metals, as co-catalysts. These materials were tested for CO_2_ photo-reduction into liquid fuels and in the production of H_2_ by photo-reforming of carbohydrates, in an attempt to try and identify the main features determining the catalytic performance.

## 2. Experimental

### 2.1. Catalyst Preparation

P25 is a commercial titanium dioxide supplied by Evonik (Essen, Germany) [39]. It was used in this form, without further adaptation.

Monometallic co-catalysts were obtained via wet impregnation over the pre-synthesized TiO_2_ and reduced under hydrogen flow at the appropriate temperature, as reported in [40]. Briefly, the selected quantity of metal precursor and titanium dioxide was added to a round flask containing distilled water. After 1 h stirring, the solution was evaporated under reduced pressure and the resulting powder was dried overnight in a conventional oven set at 105 °C. Then, the catalyst was reduced under hydrogen flow (3 mL/min, 1 atm) at high temperature (150–700 °C) in a tubular oven (Carbolite, Sheffield, UK). The reduction conditions were determined after Temperature Programmed Reduction (TPR) analysis under H_2_ flow (40 mL/min, 10 vol% H_2_/N_2_ [41]). For each sample, a heating ramp of 5 °C/min and 3 h at the selected temperature was used. The maximum reduction temperature depended on the co-catalyst and it was 150 °C for Ag, 300 °C for Pd and 700 °C for Au and Pt [40].

Bi-metallic photo-catalysts were also prepared through deposition-precipitation synthesis [41], as this deposition technique allows more precise control of the composition. In detail, 1 wt% (Au_x_Ag_y_) material was prepared by adding 0.07 mM AgCl solution (99% purity, Sigma Aldrich, Saint Louis, MO, USA), 0.03 mM HAuCl_4_ solution (99.99% purity, Sigma Aldrich) and distilled water until reaching 400 mL of volume and the desired ratio between the metals. Then, 0.10 mL of 87–90% hydrolyzed polyvinyl alcohol (PVA, 1 wt%, Sigma Aldrich) was added under vigorous stirring, followed by addition of a 0.10 M NaBH_4_ solution (99% purity, Sigma Aldrich). The molar ratio between the reducing agent and the metal was 4:1 (mol/mol). The colloidal solution was stirred for 10 min in order to stabilize the nanoparticles so formed (NPs). Then, 1 g of titanium dioxide P25 was added to the flask, followed by 0.50 mL of concentrated H_2_SO_4_ (98.0%, Sigma Aldrich). The suspension was stirred for 1 h to allow deposition of the NPs over the TiO_2_ surface, then filtered under vacuum and washed three times with water and once with methanol. The obtained powder was dried in a conventional oven at 105 °C. The same procedure was adopted for the 1 wt% (Au_x_Pt_z_) photo-catalyst, but the NaBH_4_/metal ratio was increased to 8 and no sulfuric acid was added. Photo-catalysts have been used and characterized as obtained. Metals loading was obtained by atomic absorption analysis (AAS) of the filtrate [39].

The two catalyst series were compared, but were clearly distinct. For monometallic samples a very simple procedure was used to prepare the catalysts. Impregnation is easily scalable and industrially viable, but does not allow very high dispersion at increasing loading. It was used for low loading metals addition, only. For bi-metallic samples, in order to have visible effect of the addition of the second metal and a sensitivity to variable concentrations, the overall load was increased. In order to keep dispersion safely high and deposition uniform we selected a sol immobilization procedure. Indeed, DP makes it possible to improve the control during deposition and to ensure the two different metal species are well dispersed and separated. This was essential to allow fine and controlled deposition of bi-metallic samples that were characterized by high metal loading. On the other hand, even if DP may also be used for the deposition of single metals, it was considered unnecessary, and would only add useless complexity to the preparation protocol when single metals and very low loading were planned. In such a case, the much simpler and scalable wet impregnation method was adopted.

### 2.2. Characterization

N_2_ adsorption and desorption isotherms of all samples were collected on an ASAP 2420 (Micromeritics Instrument, Norcross, GA, USA) apparatus after degassing the samples at 90 °C under vacuum (1 mm Hg) to remove water and other species adsorbed on the surface. Brunauer-Emmett-Teller (BET) linearization was used in the range 0.05–0.30 P/P° to calculate the specific surface area (SSA_BET_). Barrett-Joyner-Halenda model (BJH) and density functional theory (DFT) were used to determine pore-size distribution from the adsorption branch assuming either slit-like or cylindrical pores. CO_2_ sorption curves at the temperature of 273 K were obtained on selected samples in order to obtain the micropores distribution.

Diffuse reflectance (DRS) UV-Vis spectra were obtained in the range 200–800 nm on a Lambda 750 (PerkinElmer, Shelton, CT, USA) spectrophotometer equipped with an integrating sphere. Spectralon^®^ was used as the reflectance standard (Labsphere Inc., North Sutton, NH, USA). The sampling rate was set to 1 nm while the slit width was 2 nm. The results were processed according to the Kubelka-Munk model, using Equation (1) to transform the reflectance spectra into the absorption spectra [42,43].

(1) $$F\left(R_{\infty }\right)=\frac{{\left(1-R_{\infty }\right)}^{2}}{2R_{\infty }}$$

(F(R)hν)^1/r^ (with r = 2 or ½ for direct and indirect band gap) were plotted versus hν to determine the band gap of each sample [44].

Scanning Electron Microscopy (SEM) images and Energy Dispersive X-ray Analysis (EDX) spectra were obtained using a JSM-7900F Schottky Field Emission Scanning Electron Microscope (JEOL, Tokyo, Japan) operating at an accelerating potential of 20 kV.

X-ray diffraction (XRD) was performed through a Rigaku D III-MAX horizontal-scan powder diffractometer (Rigaku, Tokyo, Japan) using the Cu-Kα radiation and a graphite monochromator on the diffracted beam in the range 10–90°. Characteristic reflections were assigned by comparison with the standard JCPDS card, 21–1272 for anatase and 21–1276 for rutile.

### 2.3. Activity Test

All the activity tests were carried out with an innovative high-pressure stainless steel photo-reactor, previously described [41,45]. The internal reactor volume was around 1.9 L. The reactor was generally charged with 1.2 L of solution, allowing ca. 0.1 L of headspace once the cap with the lamp was placed. A medium-pressure mercury lamp with peak emission at 365 nm and measured irradiance of 157 W/m^2^ was used. Each experiment was carried out at 80 °C by recirculation of hot water in the reactor jacket and under magnetic stirring to ensure the correct mixing of the solution.

For CO_2_ photo-reduction, the reactor was usually charged with 0.031 mg/L of photo-catalyst, 1.67 g/L of sodium sulfite (which acts as hole scavenger). The pH was set to 14 using sodium hydroxide, while carbon dioxide was dissolved in the reaction media by increasing the pressure up to 20 bar.

Carbohydrate photo-reforming for hydrogen production was performed at lower pressure (4 bar of N_2_), with catalyst loading of 0.25 g/L) and with 5 g/L of glucose as HS, so far identified as providing the best conditions.

As for the tests of photoreduction of CO_2_, the maximum error on chromatographic analysis was ca. 5%. Repetition of the same test under identical conditions led to an experimental error up to 10% for both applications, increasing to a maximum observed deviation of 19% in the case of very low productivity in the gas phase for H_2_. In the case of photo-reforming, the maximum experimental error on H_2_ productivity (based on 5 reproducibility tests) was ±7.3%, while on TOC analysis was 10.2% [46].

## 3. Results and Discussion

### 3.1. Diffuse Reflactance UV-Visible (DR UV-Vis)

**Figure 1:**
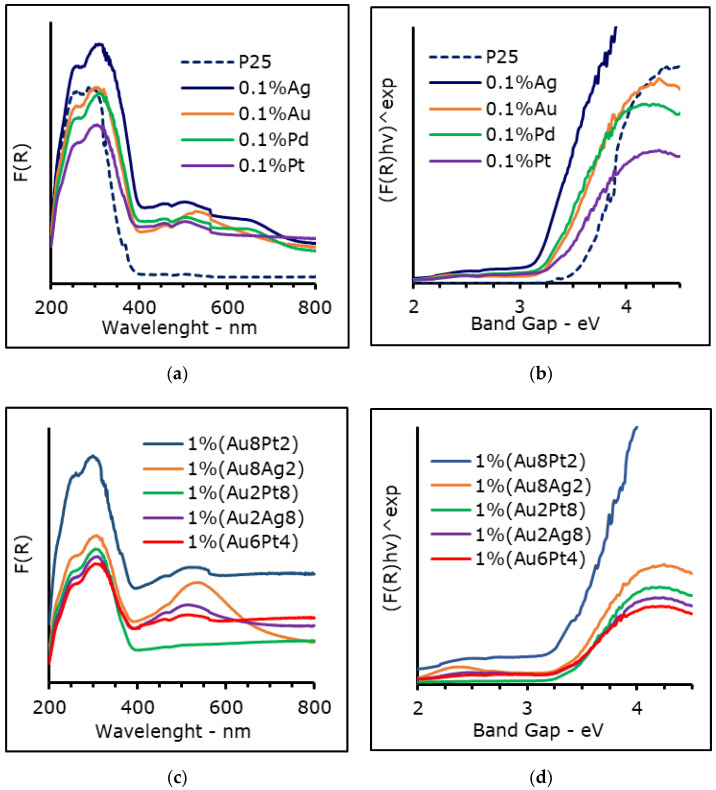
DRS UV-Vis spectra of (**a**) mono-metallic and (**c**) bi-metallic photo-catalysts and respective Kubelka-Munk transformation (r = 1/2) plotted against the BG energy (**b**,**d**). The step at 565 nm is due to instrumental reasons.

The reflectance data were reported using Kubelka-Munk and fitted through the Tauc equation to calculate both the direct and indirect transition band gap, as reported in Figure 1. The main difference between those types of transition is that for semiconductors, characterized by a direct band gap, both the absorption and the emission allow photo-excited electrons, which are usually strong. In the case of an indirect transition, the electronic relaxation between the conduction and valence bands is forbidden, and the electron-hole pair needs to change both energy and momentum, leading to a weaker emission [47]. While anatase is reported to be an indirect band gap semiconductor, rutile and brookite are not clearly identified within one of these categories [48,49,50].

The optical properties of the presented materials are coherent with the expectations, since each functionalized photo-catalyst showed a band gap lower than that of the bare P25, as reported in Table 1. In more detail, silver and platinum presented a direct BG of 3.21 eV, while for gold it was 3.20 eV, against 3.45 of pure P25 (Figure 1a–d and Figure S1). The difference is lower when looking at the indirect band gap, since from the 3.22 eV of P25 it is possible to reach values as low as 3.10 eV in the case of Ag. All these three metals also showed surface plasmonic resonance (SPR) bands, in particular the band of gold was clearly visible at 560 nm and Ag showed a peak of lower intensity around 490 nm [19,25]. On the other hand, Pt showed something similar to Ag, though its SPR bands are usually located at lower wavelengths, depending on size of the metal NPs [12]. Strong bands were observed even in the case of bi-metallic samples, in the catalysts containing gold and silver, such as 1 wt% (Au_8_Ag_2_), but not when higher loading of platinum was employed (i.e., 1 wt% (Au_2_Pt_8_)), since the curve was flat at the wavelength typical of the SPR effect [27,51,52]. In general, BG values were the same or slightly lower for bimetallic samples, depending on the composition. BG for 1 wt% (Au_2_Pt_8_) was 3.20 eV and for both 1 wt% (Au_x_Pt_z_) and 1 wt% (Au_x_Ag_y_) the direct BG was lower when the percentage of gold was reduced, while only in the case of an indirect band gap of 1% (Au_8_Ag_2_) was its value higher than that of P25.

Deposing metal nanoparticles over TiO_2_ surface is a well-known method to improve its light harvesting capability, since the metal and the semiconductor are electrically connected and the electrons can flow from or to the metal. The direction of the electron flow depends on the position of its work function in respect to the conduction band (CB) of titania [9]. For example, platinum is capable of accepting electrons, and acting as a catalytic center of the species adsorbed on its surface, while gold can inject e^-^ into the TiO_2_ semiconductor [3]. This effect can increase up to a certain limit with increasing metal load [21,41], after which a slight shielding effect by the metal predominates. A photo-catalyst loaded with 1% by weight of Au and Pt, such as 1 wt% (Au_6_Pt_4_), roughly corresponds to 0.24% mol Au and 0.16% mol Pt molar loading, which is higher than the 0.1% molar loading of the single metal co-catalyst (e.g., 0.1% mol Au). Additionally, the reduction of BG can be attributed to the partial reduction of TiO_2_ during metal reduction, which is a well-known method to adjust the BG of this semiconductor. Such a reduction can be catalyzed by the presence of the metal itself.

### 3.2. Textural Properties

**Figure 2:**
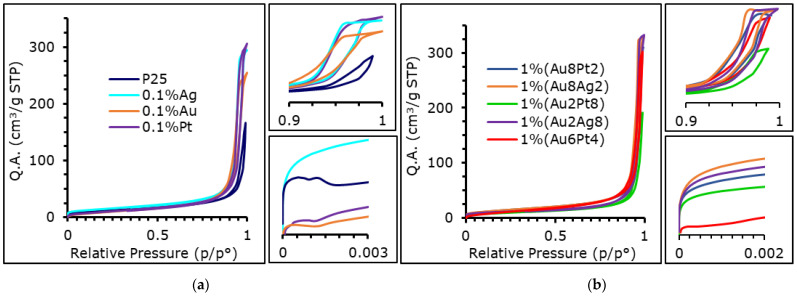
Isothermal linear plot obtained from nitrogen sorption curve of (**a**) mono-metallic and (**b**) bi-metallic photo-catalysts.

The BET surface area and pore volume were obtained from N_2_ sorption isotherms collected at a temperature of −196 °C. The results reported in Figure 2a,b show that every sample was characterized by a type II isotherm with a hysteresis loop that can be defined as H2b, characteristic of mesoporous material with a narrow distribution of pores and a wide distribution of neck size [53]. Intra-particle porosity is best described by assuming a cylindrical pore shape, while for inter-particle porosity it was more appropriate to assume slit-like pores.

The textural properties of all the samples were quite similar to each other (see Table 2). The surface area of mono-metallic samples varied between 35 and 57 m^2^/g. The lowest value was attained with Pt. The BET SSA of the bi-metallic samples was generally equal to that of P25 TiO_2_, with the exception of 1 wt% (Au_x_Pt_y_), for which a decrease of surface area and porosity were evident. The same behavior was observed in the monometallic sample, since 1 mol% Pt showed quite a low surface area (33 m^2^/g). In general, however, the effect of the addition of metals was not impactful on the textural properties of the materials. Furthermore, while the reduction process for the mono-metallic samples occurred at high temperature and, in principle, can cause the collapse of pores and a loss in terms of surface area [11], bi-metallic samples were chemically reduced in milder conditions. However, the thermal treatment, even at the highest temperature, did not significantly affect the porosity of the samples. This behavior is probably due to the high temperature preparation of the native titania P25, which confers significant thermal resistance to sintering, when compared to titania obtained by hydrolysis (e.g., starting from titanium isopropoxide) [54].

Several photo-catalysts, including P25, 0.1 mol% Pt, 0.1 mol%Au and 1 wt% (Au_2_Pt_8_) were characterized by a strong interaction with nitrogen at the beginning of the adsorption process (see in the zoom in the 0–0.002 P/P°).

P25 showed some inter-particle microporosity (these materials are usually constituted by dense nanospheres, possibly condensing by leaving cavities between adjacent particles) that was only still present, after surface modification, for 0.1% Ag and Au_x_Ag_y_ samples (Supplementary Materials Figure S2). This behavior may be linked to the strong metal-support interaction (SMSI) that occurs between Pt and titania when the material undergoes a reduction process; especially when reduction is performed at high temperature and with a strong reducing agent (H_2_ and NaBH_4_ in our case) [10]. However, the t-plot method is not suitable to describe each sample, as in the case of P25, 0.1 mol% Pd, 0.1 mol% Ag. For these catalysts, the adsorption took place at very low pressure, which is linked to ultra-microporosity, that cannot be correctly quantified by N_2_ adsorption. Moreover, other models, such as Dubinin-Astakhov, are not really applicable since they are more suitable for carbon materials that strongly interact with the quadrupole moment of nitrogen [55,56]. The strong metal-support interaction mentioned above, which is observed in some cases, is likely to interfere with the adsorption process. Furthermore, SMSI is less likely to occur for gold and silver, which deposit with larger particles compared to Pt [57,58].

Lastly, the CO_2_ sorption curves were obtained for selected photo-catalysts and the results are consistent with those obtained with N_2_.

### 3.3. Scanning Electron Microscopy and Energy-Dispersive X-ray (SEM-EDX)

**Figure 3:**
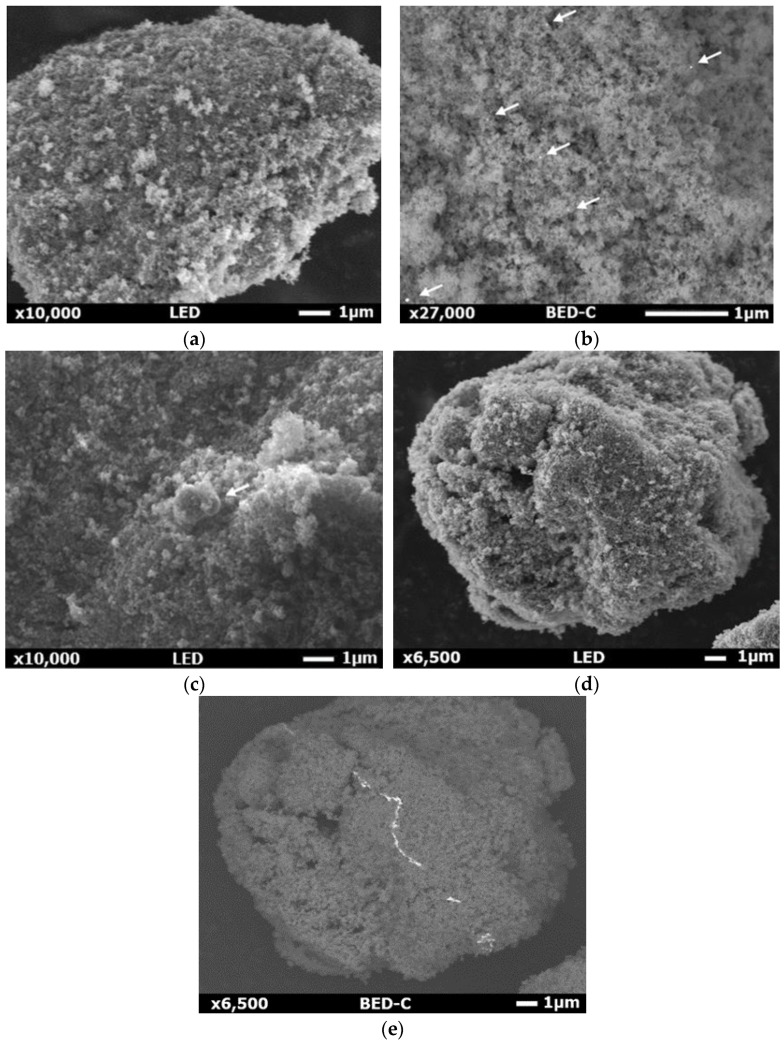
SEM images of (**a**) and (**b**) 0.1 mol% Au/P25, (**c**) 0.1 mol% Pt/P25 and (**d**) and (**e**) 1 wt% (Au_6_Pt_4_)/P25. (**b**) and (**e**) have been taken using backscatter detector (BED-C).

SEM-EDX analysis was performed on selected materials and the results are reported in Figure 3 and Figure S3 (see Supplementary Information File). All the samples were constituted of a uniform array of titania nanoparticles ca. 20 nm in size. It was observed that the morphology of the NPs did not change after addition of metals, nor did it change after calcination at high temperature (when relevant for monometallic samples). This further confirms the very good thermal resistance of titania P25.

The composition was checked locally by EDX, but because of the low metal load and the high metal dispersion, it was necessary to increase the acquisition time up to 12 min. The metal NPs were observed with difficulty through EDX mapping (see e.g., Figure S3 for one of the most visible materials), though the presence of the metal on the catalyst surface was confirmed to be in the desired loading by XPS analysis (*vide infra*).

The formation of a metal nanocluster of tens of nanometer was already reported, but usually concerned higher metal loading than that present in the here investigated samples [27,59]. Elemental mapping of the 1 wt% (Au_6_Pt_4_) sample highlighted only a small region of the nanoparticles in which both Au and Pt were deposited, but it was not possible to identify any metal NPs on its surface. For Au_8_Pt_2_ very small particles were visible at high magnification of 70,000×, while the backscatter detector (BED-C) allowed us to see smaller and more abundant NPs on the 0.1 mol% Au sample. Similar results were obtained with 1 wt% (Au_x_Ag_y_) photo-catalysts (Figure S3 in Supplementary Information), since NPs were observed on the particle surface when the backscatter detector was used. Tentatively, it may be considered that bigger particles, thus more visible with EDX mapping and with a back scattering detector, were somehow formed with Pt, both when added alone or with another metal.

So far, it was not possible to identify if the co-catalysts were deposited in the form of alloy or as a separate single metal NP. Apparently, the location of Au and Pt in EDX maps (Figure S3) appear distinct from each other, suggesting they do not form an extensive alloy.

### 3.4. X-ray Photoelectron Spectroscopy (XPS)

**Figure 4:**
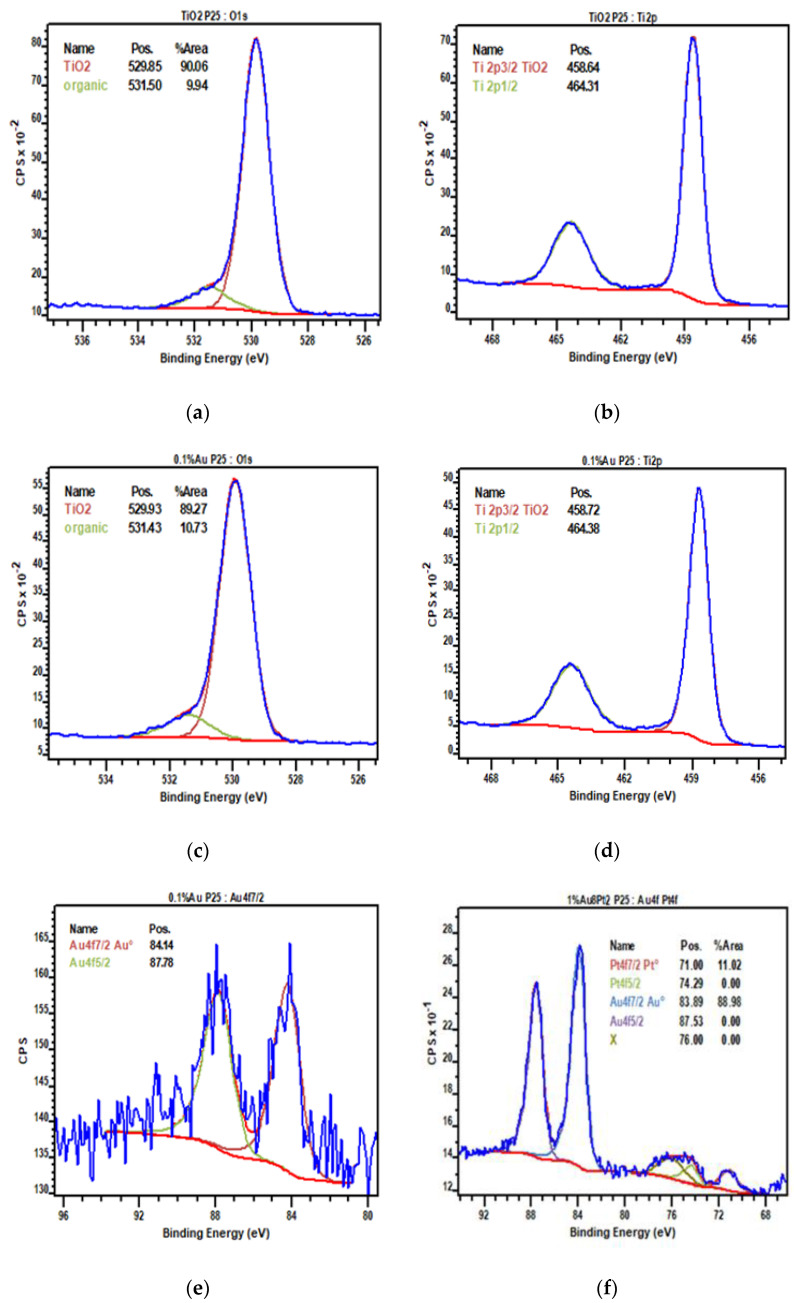
XPS spectra of selected photo−catalyst: (**a**) O1s P25; (**b**) Ti2p P25; (**c**) O1s 0.1 mol% Au; (**d**) Ti2p 0.1 mol% Au; (**e**) Au4f 0.1 mol% Au; (**f**) Au4f and Pt4f 1 wt% (Au_8_Pt_2_).

XPS spectra of selected photo-catalysts are illustrated in Figure 4 and in the Supplementary Information file (Figure S4). All the analyzed samples contained organic impurities (up to 11.15%) that have been associated with various organic moieties (C-C, C-H, C-O, C-C=O, O=C-O), based on the type of bonds evidenced by deconvolution of the peaks. The XPS peak intensity areas were obtained after subtracting a Shirley type background, since the component from the inelastic scattered electrons of the Ti3p and Ti3s peaks appear in the same region of Pt4f and Au4f core levels [60,61,62]. In general, the metal deposition process does not significantly influence the shape and intensity of the O1s and the Ti2p peaks of titania; the metals are probably too diluted and, as an example, the spectrum of 0.1 mol% Au is barely distinguishable from that of P25 (Figure 4a), at least for what concerns the Ti and O regions [63].

The main peak at 459 eV is due to Ti^4+^ species and overlaps with the small peak assigned to Ti^3+^ that can be found at 457 eV, especially in the spectra of 1 wt% (Au_6_Pt_4_) and 1 wt% (Au_8_Pt_2_). The presence of Ti^3+^ is due to the reduction process of the metallic active phase of the catalysts, which leads to partial reduction of the Ti^4+^ ion on the photo-catalyst surface, thus modifying its electronic properties and BG values. Ti^4+^ reduction can be catalyzed by the metal itself, but due to the low amount of Au and Pt, the Ti^3+^ peak is invisible, not visible on the Ti2p spectrum.

Regarding the metals, they are present in the reduced state. The peaks associated with Pt^0^ and Au^0^ are characterized by a low signal-to-noise ratio (Figure 4b), while signals are more intense in the case of bi-metallic samples, since the loading is higher than in mono-metallic catalysts. The characteristic peaks of gold were identified at 84 eV (4f7/2) and 88 eV (4f5/2). For platinum, peaks were detected at 71 eV (4f7/2) and 74 eV (4f5/2), while in the literature Pt^2+^ peaks are usually located at a slightly higher BE (ca. 72 and 75 eV [64,65,66]).

As a general observation, the active material was revealed to be stable after the synthesis and can be stored without undergoing significant oxidation. This is positive, because with such metals the presence of oxides may reduce the activity if not added on purpose and by controlling the distribution [67]. In addition, most of the samples also contained traces of aluminum oxide (around 76 eV). Lastly, according to the area of the peaks, it was found that the metal loading on the extreme surface was higher than 0.1% for the mono-metallic photo-catalysts (i.e., Au and Pt) and even two times more for the bi-metallic materials (Table 3) with the exception of 1 wt% (Au_8_Pt_2_). This over-concentration at the surface is expected, since the loading procedure deposits the metal only at the surface. Differently, in the case of 1 wt% (Au_6_Pt_4_) the ratio between the two elements is 2:1 rather than 3:2, while it becomes 7:1 for Au_8_Pt_2_ instead of the theoretical value of 4:1. So, there is a small portion of the platinum that is not visible at the surface. The missing platinum is likely to hide beneath the surface of the TiO_2_ support, or be covered with gold, as strong metal-support interactions (SMSI) may develop during the reduction process and the Pt particle may sink into the support and be covered by titania [11,18]. Au is less likely to develop this type of interaction [11,57,58].

### 3.5. X-ray Diffraction (XRD)

**Figure 5:**
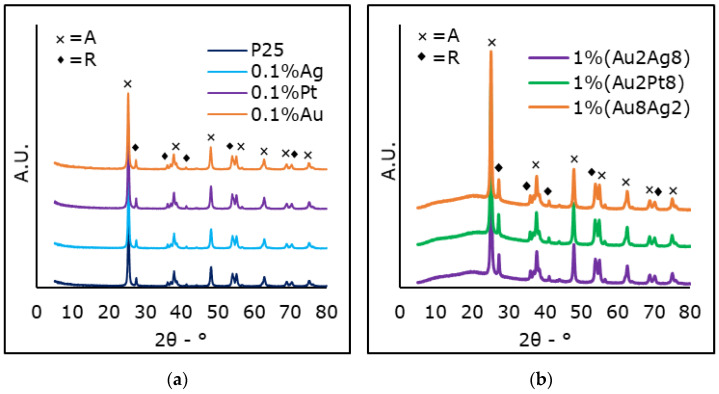
XRD spectra of bare P25 compared with (**a**) mono and (**b**) selected bi−metallic photo-catalysts. “A” stands for Anatase and “R” refers to Rutile.

XRD spectra have been obtained for every photo-catalyst (Table 4 and Figure 5). As the metal loading was very small, no characteristic peaks related to metallic phases could be detected in diffractograms (Figure 5) [25]. The diffractograms evidenced only the characteristic phases of titania, anatase and rutile, which are well known for the P25 raw material. The reduction process did not have a significant impact on the crystalline phase composition of the bare TiO_2_ P25, as the anatase/rutile ratio of modified photo-catalysts did not differ from that of bare titania (ca. 70–80% anatase and 20–30% rutile), according to relative intensities of anatase main peak (25.4°) and rutile (27.4°). Based on the literature, once the amorphous TiO_2_ or the anatase phase is heated above 400 °C it starts to convert into the more stable rutile phase [21,27]. However, it has already been reported that P25 is mostly unaffected by thermal cycles that are necessary in order to reduce the noble metals added by impregnation [68]. In that case, gold delays the transformation of anatase into rutile [54].

Finally, crystallite size was calculated applying the Scherrer equation to the main reflection of each phase [69]. Anatase had, in general, lower crystal size than rutile. The size slightly increased upon thermal treatment following impregnation in the case of the mono-metallic samples. On the contrary, for the bi-metallic samples, chemical reduction was achieved with NaBH_4_ under ambient conditions. This did not alter the size of anatase crystals and even slightly decreased the size of the rutile phase. This point was interpreted by the formation of a larger quantity of defects (formation of oxygen vacancies) when reducing the rutile phase. This is visible for rutile, since it is more reducible than anatase [70].

### 3.6. Photo-Catalytic Tests

The photo-catalysts analyzed in this work have been used for various applications, including CO_2_ photo-reduction, and photo-reforming of organics for hydrogen production. Both these reactions represent photo-reduction processes, which exploit electrons photo-promoted in the conduction band of the semiconductor upon irradiation to reduce the target reactants, i.e., CO_2_ into reduced molecules, such as HCOOH, HCHO, CH_3_OH and CH_4_, or H^+^ to H_2_ in the case of photo-reforming. The complementary oxidation half reaction is achieved through the holes, which react with electron donating molecules, i.e., sodium sulfite in the case of CO_2_ photoreduction, and glucose (or other sacrificial organics in water) in the case of photo-reforming.

Blank experiments [41] have been carried out for the photo-reduction of CO_2_ by irradiating a solution at 80 °C and 8 bar, first without any catalyst nor HS and later with HS (1.68 g/L) but without a catalyst. In both cases, nil productivity, either in gas or liquid phases, were observed. Another test at the same temperature and pressure, using bare P25 under dark conditions, was performed without observing significant productivity. It cannot be excluded that some adsorption of CO_2_ may occur, though not quantifiable, due to its high concentration in the liquid phase and limited catalyst amount.

Tests in dark conditions were also performed for the photo-reforming of glucose, extensively described in [46], confirming that irradiation is necessary to obtain the products. Moreover, a negligible conversion was obtained by using the light source, but without a catalyst and/or HS.

#### 3.6.1. Photo-Reduction of CO_2_

**Figure 6:**
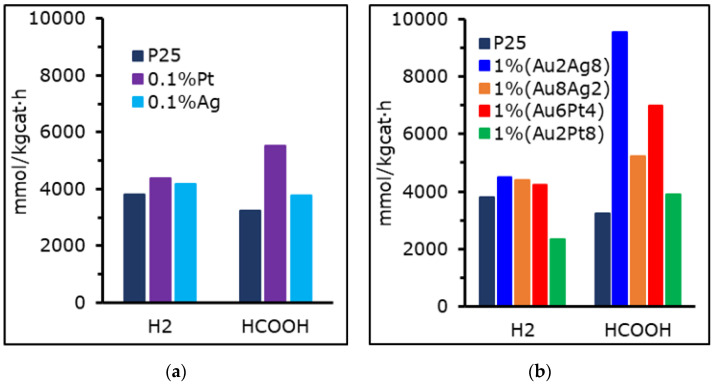
Productivity of HCOOH and H_2_ for (**a**) mono−metallic and (**b**) bi−metallic photo-catalysts from CO_2_ photo-reduction activity tests performed at 8 bar and pH = 14.

Figure 6 illustrates some of the most promising photo-catalysts employed for the conversion and valorization of carbon dioxide, which have also been reported elsewhere [41,71,72]. The most active material, at least in terms of formic acid productivity, is the bi-metallic photo-catalyst 1 wt% (Au_2_Ag_8_), leading to 9.5 mol/kg_cat_ h (±5%) and outperforming the non-promoted titania P25 (3.2 mol/kg_cat_ h (±5%)). Formic acid is used here as a key performance indicator to evaluate the activity, due to its high productivity in the selected conditions (basic pH, high operating pressure). On the other hand, hydrogen is also likely to be produced once the hole scavenger (sodium sulfite) conversion reaches 100%, so the organic products formed by photo-reduction of CO_2_ act as HS themselves. H_2_ productivity is on average 4.0 mol/kg_cat_ h (±7.3%) for all the materials here compared, though this value is almost halved for 1 wt% (Au_2_Pt_8_). Interestingly, this catalyst has quite a low surface area (33 m^2^/g), despite being prepared via DP, if compared to the others, and a low total pore volume comparable with that of bare P25. So, this may negatively affect its performance, despite its having a low band gap.

All the catalysts have a relatively similar surface area, in the range of the native P25 material, while some of the photo-catalysts reported in literature are characterized by a surface area greater than the present ones, usually >100 m^2^/g, due to the different preparation techniques [25,59]. Regarding the textural properties, for 0.1 mol% Pt prepared through wet impregnation, the SSA value was quite a lot lower than for P25 TiO_2_ (34.9 m^2^/g vs. 41.4 m^2^/g) while 1 wt% (Au_2_Ag_8_) has 40.7 m^2^/g and it is higher in case of 1 wt% (Au_6_Pt_4_) (44.6 m^2^/g). What differs is that for all of them the total pore volume is doubled with respect to P25, but only in the case of Au_2_Ag_8_ was the micro porosity maintained after the treatment.

Regarding the BG, all the best three catalysts (i.e., 1 wt% (Au_2_Ag_8_), 1 wt% (Au_6_Pt_4_) and 0.1 mol% Pt) have a low direct band gap, since this value is 3.21 eV for both the bi-metallic and the mono-metallic samples, while the bare titania P25 has BG = 3.45 eV and exploits the worst activity of the selection. The UV lamp employed has its peak emission at 365 nm but the light is actually polychromatic. Being a medium-pressure mercury-type lamp, a lower band gap increases the amount of energy harvested from that source [12,13,47].

The present results show significant improvements with respect to most literature, since they demonstrate a photo-reactor setup unique in literature, operating at high pressure and high temperature (relative to the conventional conditions adopted in photocatalysis). Certain considerations can be made concerning the activity of our photo-active material. Table 5 reports the performance of several photo-catalysts based on P25 titanium dioxide and employed for the CO_2_ photo-reduction. As shown, the main species released during the treatment and their productivity mainly consist of liquid products (formic acid, methanol, formaldehyde) and gaseous ones (hydrogen, carbon monoxide, methane). In our setup, the main products are formic acid and, on demand, hydrogen, which comes from the photo-reforming of the organics formed from CO_2_, rather than directly from the water splitting. Indeed, in the selected conditions (24 h of reaction) the HS scavenger was always fully consumed, and, once this happens, the organics accumulated in the solution are reformed to obtain hydrogen [73]. Kaneco et al. [74] reported a very low productivity of these compounds when working at high pressure and in the presence of an organic hole scavenger. However, in that case a very high concentration of unmodified titania had been used, and it is likely that the system was limited by the shadowing effect. On the other hand, using our modified photo-catalysts in very low concentration and at high pressure leads to good performance in terms of organics productivity.

#### 3.6.2. Photo-Reforming

**Figure 7:**
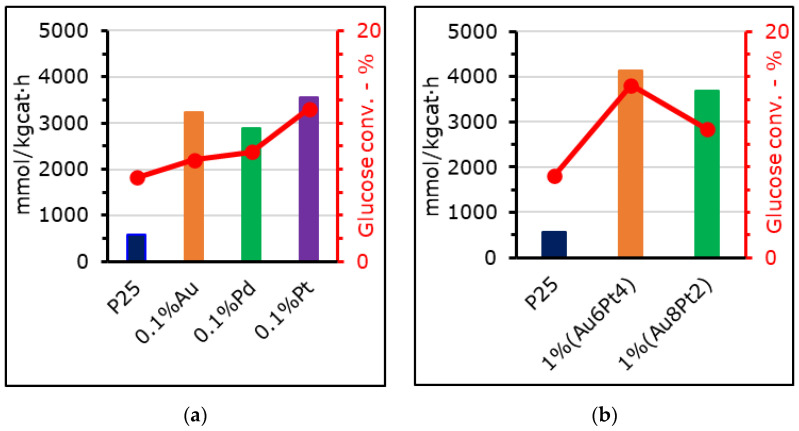
Hydrogen productivities through glucose photo−reforming achieved with 0.250 g/L of photo-catalyst, 5 g/L of glucose at pH = 6.5, 4 h test with medium pressure UV lamp. (**a**) mono-metallic and (**b**) bi-metallic photo-catalysts.

The same photo-catalysts were used in the photo-reforming of glucose for H_2_ production. Selected results are reported in Figure 7, where the performance of the benchmark P25 TiO_2_ is compared to both the mono- and bi-metallic co-catalysts.

Glucose was selected as an HS to assess the activity of the photo-catalysts to perform reduction of water to hydrogen when a sacrificial electron donor is added to the mixture. Indeed, without the HS the performance of the system is limited by the slow evolution of oxygen, while glucose offers a way to rapidly quench the holes (direct oxidation) or the hydroxyl radicals produced by the photo-catalyst (indirect oxidation) [7,76,77].

The 1 wt% (Au_6_Pt_4_) was able to achieve hydrogen productivity of 4.1 mol/kg_cat_ h (±7.3%) while 0.1 mol% Pd reached only 2.9 mol/kg_cat_ h (±7.3%) and the benchmark P25 0.57 mol/kg_cat_ h (±7.3%). Gas phase byproducts were also formed, such as CO_2_, CO, CH_4_ and C2 compounds (ethane and ethylene), in concentrations far lower than that of H_2_. The addition of the metal was essential for this application, due to its electron trapping effect, which allows improvement of the electron-hole separation. This prevents dissipative charge recombination and improves overall productivity.

Glucose conversion never reached values higher than 15 ± 0.12%) (1% (Au_6_Pt_4_). However, this is in line with the performance of similar materials found in literature [38].

Hydrogen evolution continuously rises during the photo-reforming treatment, as reported in Figure S5.

However, again, if looking at the BG values they seem to be strictly related to the photo-catalyst activity, since 0.1 mol% Pd was characterized by a direct BG of 3.33 eV and achieved 23% lower productivity than 0.1 mol% Pt (BG = 3.21 eV), despite having a greater surface area. On the other hand, 0.1 mol% Au had a BG of 3.2 eV, but also showed a SPR band at 560 nm, which positively influenced the photo-generation of electrons.

Gallo et al. [27] reported the photo-reforming of ethanol using a similar catalyst based on titania and loaded with 0.5 wt% of gold and 0.5% of platinum, obtaining hydrogen productivity as high as 7500 mmol/kg_cat_ h. Our bi-metallic sample of 1 wt% (Au_6_Pt_4_) was the most similar in terms of composition, but its productivity was lower, since it reached 4100 mmol/kg_cat_ h (±7.3%). However, it is necessary to consider that our photo-catalyst concentration was eight times lower and, in a broader context, the setup configuration was different. In particular, the necessity to operate at 4 bar in our experimental assembly, due to sampling methodology, disfavors product evolution in the gas phase, but most of all, a much simpler HS was used in the reported reference as ethanol, with respect to glucose, here used as a more complex substrate, representative of milk and food waste waters. On the other hand, the mono-metallic catalysts, like 0.1 mol% Pt, performed quite well if compared to analogue materials that can be found in literature (see Table 6) [21,37,38]. Indeed, Vaiano et al. reported photo-reforming of glucose under UVA irradiation achieving an H_2_ productivity of about 200 mmol/kg_cat_ h when using a titania-based photo-catalyst loaded with 0.5 wt% of palladium. In our case, productivity using a similar but less loaded catalyst (i.e., 0.1 mol% Pd) allowed us to achieve a productivity fifteen times higher.

## 4. Conclusions

The major contribution to the photo-catalytic activity is due to the transition from the valence to the conduction band of the semiconductor, i.e., the direct band gap. The materials with the lowest BG, such as 1 wt% (Au_2_Ag_8_), 1 wt% (Au_6_Pt_4_), and 0.1 mol% Pt, reached the highest productivities in both photo-reduction reactions. For instance, they outperformed the benchmark P25 by, respectively, 295%, 215% and 70% in the CO_2_ photo-reduction, and by 722% and 622%, for photo-reforming process. Specifically, 9.5 mol HCOOH/h kg_cat_ + 4.5 mol H_2_/h kg_cat_ and 4.1 mol H_2_/h kg_cat_ were achieved for CO_2_ photoreduction and glucose photo-reforming, respectively. However, the improved activity of the catalytic material cannot be entirely explained by the lower band gap; indeed, poor performances were encountered when either the surface area was low or the total pore volume substantially decreased after surface functionalization of the catalyst, like in the case of 1 wt% (Au_2_Pt_8_)/P25. Very low noble metals loading with high dispersion is sufficient for the scope of charge separation and to boost catalyst performance.

As a general observation, it is difficult to compare the performance of the catalysts prepared with that of other materials previously reported in the literature. The reaction conditions are very different (especially as regards the pressure in the reactor and the quantity of catalyst). Using low amounts of noble metal is surely beneficial for the economics of the reaction, since the cost of the photo-catalyst is significantly impacted by the nature and the loading of the metal. The challenge in prospective work on such types of materials is the characterization procedure, since extremely low metal loading and high NPs dispersion reduce the number of characterization techniques capable of giving structural insights into these catalysts.

## Figures and Tables

**Table 1:** Band gap values (direct and indirect) of selected photo-catalyst obtained by reflectance measurements and Kubelka-Munk transformation.

Photo-Catalyst	Direct Band Gap-eV	Indirect Band Gap-eV
P25	3.45	3.22
0.1 mol% Pd/P25	3.33	3.14
0.1 mol% Au/P25	3.20	3.14
0.1 mol% Pt/P25	3.21	3.18
0.1 mol% Ag/P25	3.21	3.10
1 wt% (Au_8_Pt_2_)/P25	3.22	3.19
1 wt% (Au_6_Pt_4_)/P25	3.21	3.22
1 wt% (Au_2_Pt_8_)/P25	3.20	3.19
1 wt% (Au_8_Ag_2_)/P25	3.22	3.24
1 wt% (Au_2_Ag_8_)/P25	3.21	3.21

**Table 2:** Microstructural properties of the samples derived by N_2_ sorption isotherms at −196 °C.

Photo-Catalyst	BET SSA–m^2^/g	Total Pore Volume–cm^3^/g	t-Plot Micropore Volume–cm^3^/g	BJH Adsorption Pore Width–nm
P25	47	0.257	/	35
0.1 mol% Pd/P25	57	0.511	/	39
0.1 mol% Au/P25	38	0.394	/	32
0.1 mol% Pt/P25	35	0.474	/	38
0.1 mol% Ag/P25	53	0.455	0.00039	34
1 wt% (Au_8_Pt_2_)/P25	52	0.480	/	42
1 wt% (Au_6_Pt_4_)/P25	45	0.467	/	38
1 wt% (Au_2_Pt_8_)/P25	33	0.296	/	36
1 wt% (Au_8_Ag_2_)/P25	51	0.509	/	43
1 wt% (Au2Ag8)/P25	41	0.513	0.00310	51

**Table 3:** Theoretical composition vs. abundance calculated through XPS of selected photo-catalysts.

	Theoretical Bulk Metal Loading−mol%		XPS Loading−mol%	
Photo-Catalyst	Au	Pt	Au	Pt
0.1 mol% Au/P25	0.1	/	0.143	/
0.1 mol% Pt/P25	/	0.1	/	0.126
1 wt% (Au_8_Pt_2_)/P25	0.32	0.08	0.672	0.089
1 wt% (Au_6_Pt_4_)/P25	0.24	0.16	0.538	0.276

**Table 4:** XRD analysis results of mono and bi-metallic photocatalysts.

Photo-Catalyst	Anatase/Rutile-%	Crystallite Size
P25	78/22	15(A)/26(R)
0.1 mol% Au/P25	78/22	18(A)/28(R)
0.1 mol% Pt/P25	77/23	18(A)/28(R)
0.1 mol% Ag/P25	70/30	18(A)/28(R)
1 wt% (Au_2_Pt_8_)/P25	73/27	16(A)/25(R)
1 wt% (Au_2_Ag_8_)/P25	76/24	16(A)/24(R)
1 wt% (Au_8_Ag_2_)/P25	73/27	16(A)/22(R)

**Table 5:** Performance in the CO_2_ photo-reduction of different photo-catalysts reported in literature.

Cat.	[Cat.] mg/L	Light Source	HS	Conditions	Products mmol/kg_cat_ h	Ref.
0.1 mol% Pt	31	UVA-157 W/m^2^	Na_2_SO_3_	80 °C; 8 bar; pH 14	HCOOH-9500 H_2_-4500	This work
1 wt% (Au_2_Ag_8_)	31	UVA-157 W/m^2^	Na_2_SO_3_	80 °C; 8 bar; pH 14	HCOOH-5500 H_2_-4400	This work
2 wt% Au	5000	Xe–620 W/m^2^	i-PrOH	rt; 28 bar	CH_4_-0.5 HCOOH-<0.1	[74]
1.5 wt% Pt	287	UVC-8 W	none	rt	CH_4_-1.25 H_2_-1 CO-0.58	[59]
2 wt% Cu	100	UVC	none	rt; pH 13	CH_3_OH-23	[33]
3 wt% CuO	1000	UVC–6 W	none	rt; pH 13	CH_3_OH-230 HCOOH-0.5 HCHO-0.2	[75]

**Table 6:** Performance in the glucose photo-reforming of different photo-catalysts reported in literature.

Cat.	[Cat.] mg/L	Light Source	HS	Conditions	Products mmol/kg_cat_ h	Ref.
0.1 mol% Pt	250	UVA-157 W/m^2^	glucose	80 °C; 5 bar; pH 6.5	H_2_-3500	This work
1 wt% (Au_6_Pt_4_)	250	UVA-157 W/m^2^	glucose	80 °C; 5 bar; pH 6.5	H_2_-4100	This work
0.5 wt% Pd	500	UVA–10 W	glucose	rt; 1 bar	H_2_-208	[38]
0.2 wt% Au	600	Sunlight–145,000 lux	CH_3_OH	N/D	H_2_-400	[21]
0.5 wt% Au+0.5 wt% Pt	2000	UVA–125 W	CH_3_CH_2_OH	N/D	H_2_-7500	[27]
0.5 wt% Pt	1300	Xe–450 W	glycerol	40 °C; pH 7	H_2_-375	[37]

## Data Availability

Not applicable.

## Supplementary Materials

The following supporting information can be downloaded at: https://www.mdpi.com/article/10.3390/ma15082915/s1, Figure S1: Reflectance spectra; Figure S2: Average pore size distribution; Figure S3: Additional SEM and EDX images; Figure S4: Full XPS spectra.
