# Supplementary material for: Low Metal Loading (Au, Ag, Pt, Pd) Photo-Catalysts Supported on TiO2 for Renewable Processes
Source: Materials (Basel). 2022 Apr 15;15(8):2915. doi: 10.3390/ma15082915 (PMC9031976; doi:10.3390/ma15082915)
Supplement: Supplementary file 1 [file materials-15-02915-s001.zip › materials-1572680-supplementary.pdf]

materials

Supplementary Information

Low Metal Loading (Au, Ag, Pt, Pd) Photo-Catalysts Supported on TiO_2 for Renewable Processes

Francesco Conte [1], Ilenia Rossetti [1,*], Gianguido Ramis [2], Cyril Vaulot [3], Samar Hajjar-Garreau [3] and Simona Bennici [3,*]

[1] Chemical Plants and Industrial Chemistry Group, INSTM Unit Milano-Università, Dip. Chimica, CNR-ISTM, Università degli Studi di Milano, via C. Golgi 19, 20133 Milan, Italy; francesco.conte@unimi.it

[2] INSTM Unit Genova, Dip. Ing. Chimica, Civile ed Ambientale, Università degli Studi di Genova, via all'Opera Pia 15A, 16145 Genoa, Italy; gianguidoramis@unige.it

[3] Institut de Science des Materiaux, CNRS, IS2M UMR 7361, Université de Haute-Alsace, F-68100 Mulhouse, France; cyril.vaulot@uha.fr (C.V.); samar.hajjar@uha.fr (S.H.-G.)

* Correspondence: ilenia.rossetti@unimi.it (I.R.); simona.bennici@uha.fr (S.B.); Tel.: +39-(0)2-5031-4059 (I.R.); +33-(0)3-8933-6729 (S.B.)

1. UV-Vis Spectra

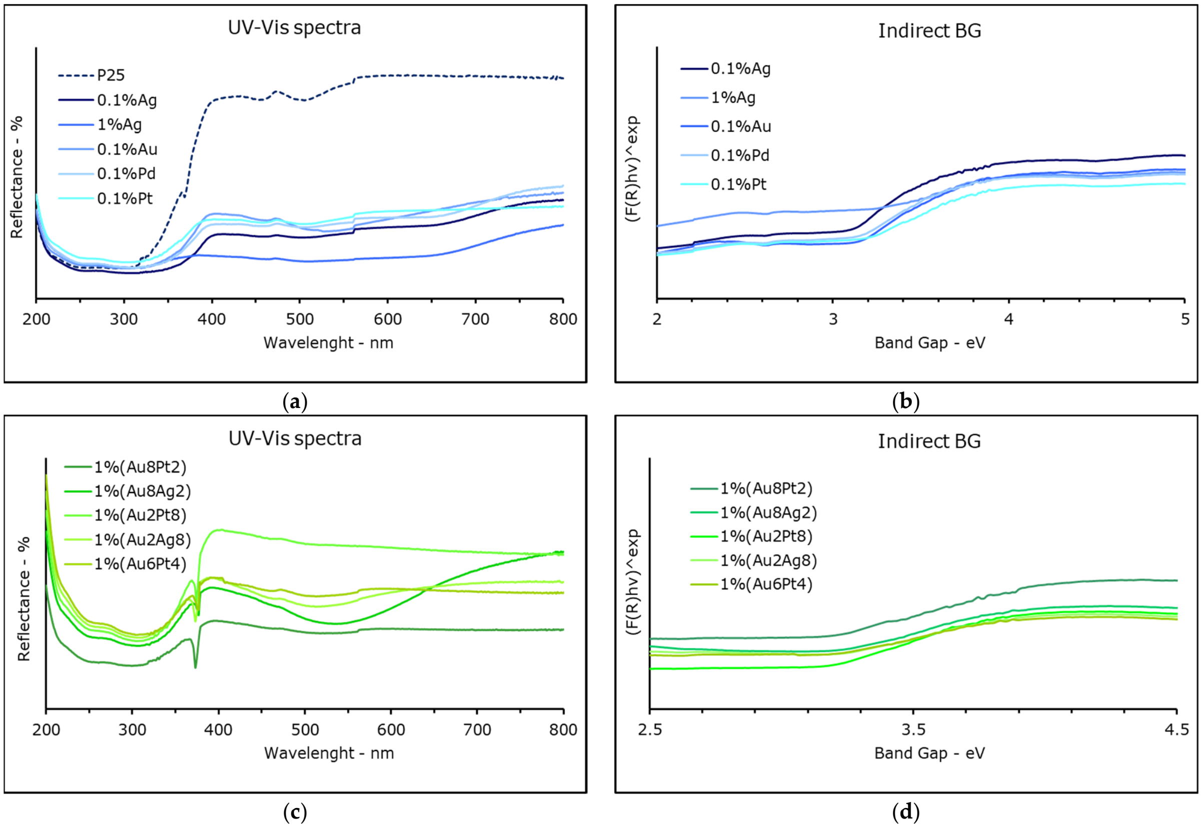

Figure S1. Reflectance spectra of (a) mono-metallic (c) bi-metallic photo-catalysts and Kubelka-Munk transformation with y = 0.5: (b) mono-metallic and (d) bi-metallic photo-catalysts.

2. BET

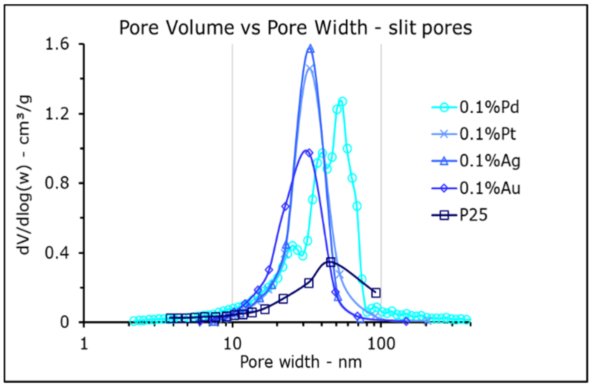

(**a**)

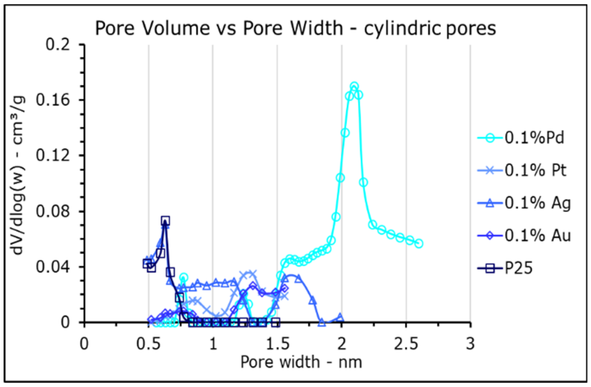

(**b**)

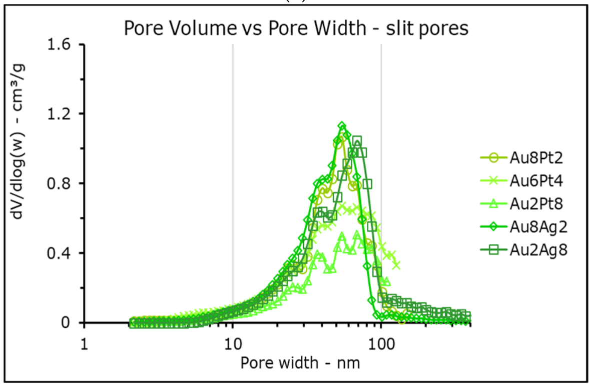

(**c**)

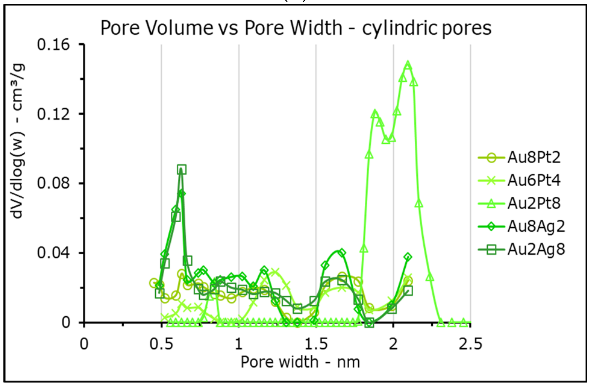

(**d**)

Figure S2. Average pose size distribution: (**a**,**c**) mono-metallic and (**b**,**d**) bi-metallic photo-catalysts.

3. SEM-EDX

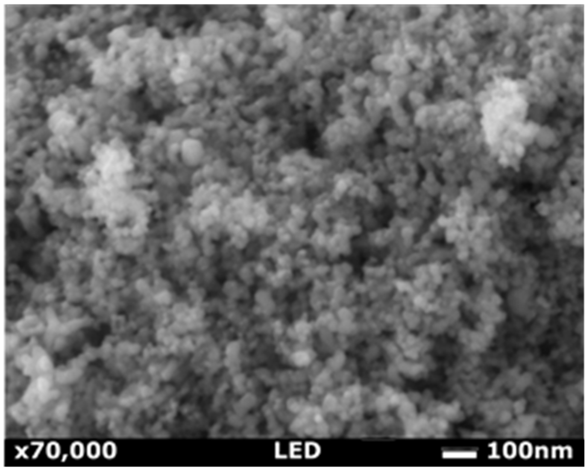

(**a**)

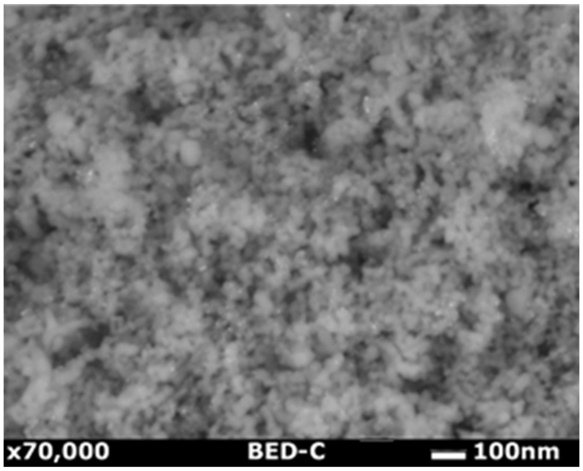

(**b**)

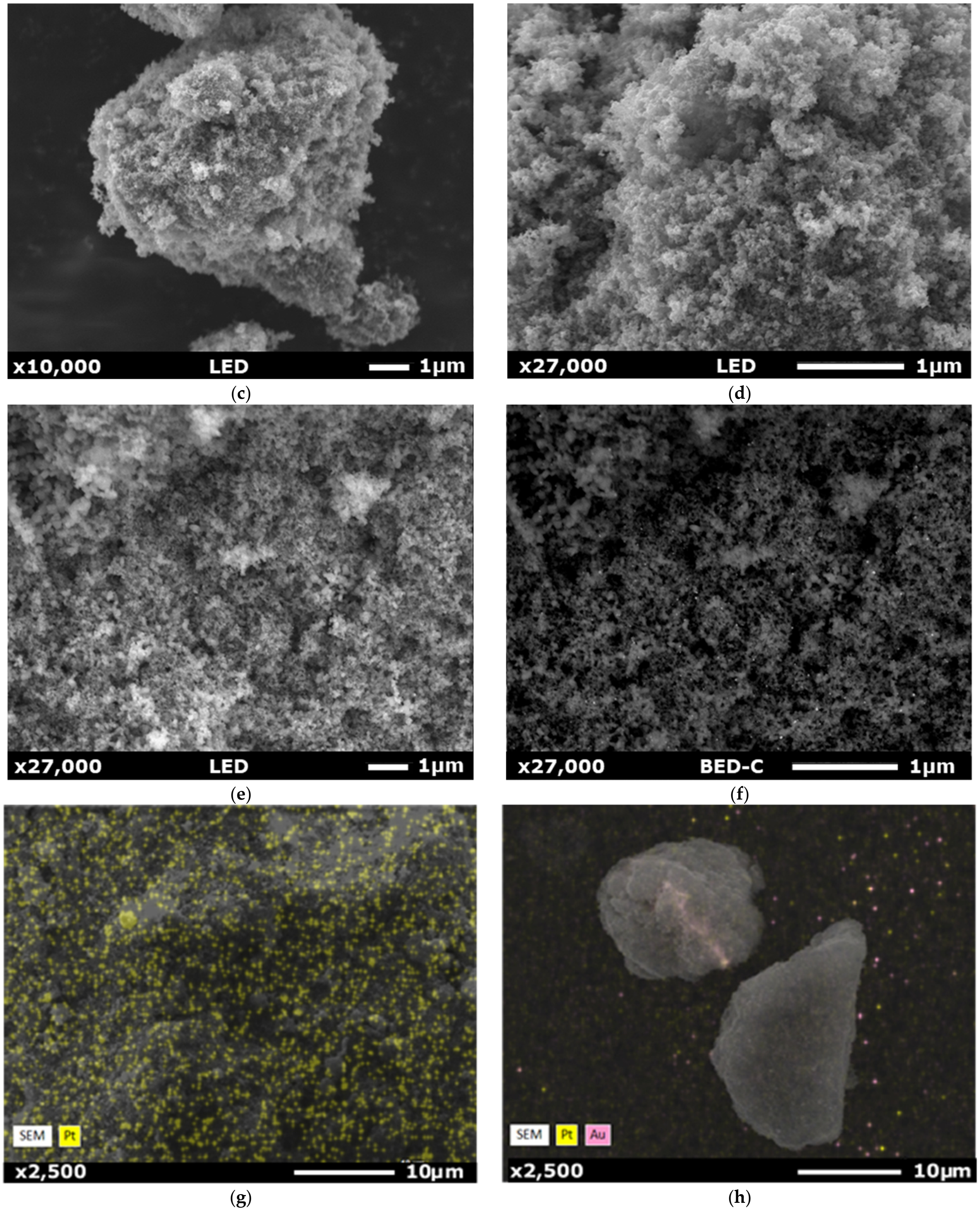

Figure S3. Additional SEM images: (**a**,**b**) 1%wt(Au_8Pt_2), (**c**,**d**) 0.1%molAg, (**e**,**f**) 01%wt(Au_2Ag_8). EDX spectra: (**g**) 0.1%molPt and (**h**) 1%wt(Au_6Pt_4). Figures (**g**,**h**) ere reprinted under the Creative Commons licence from [71].

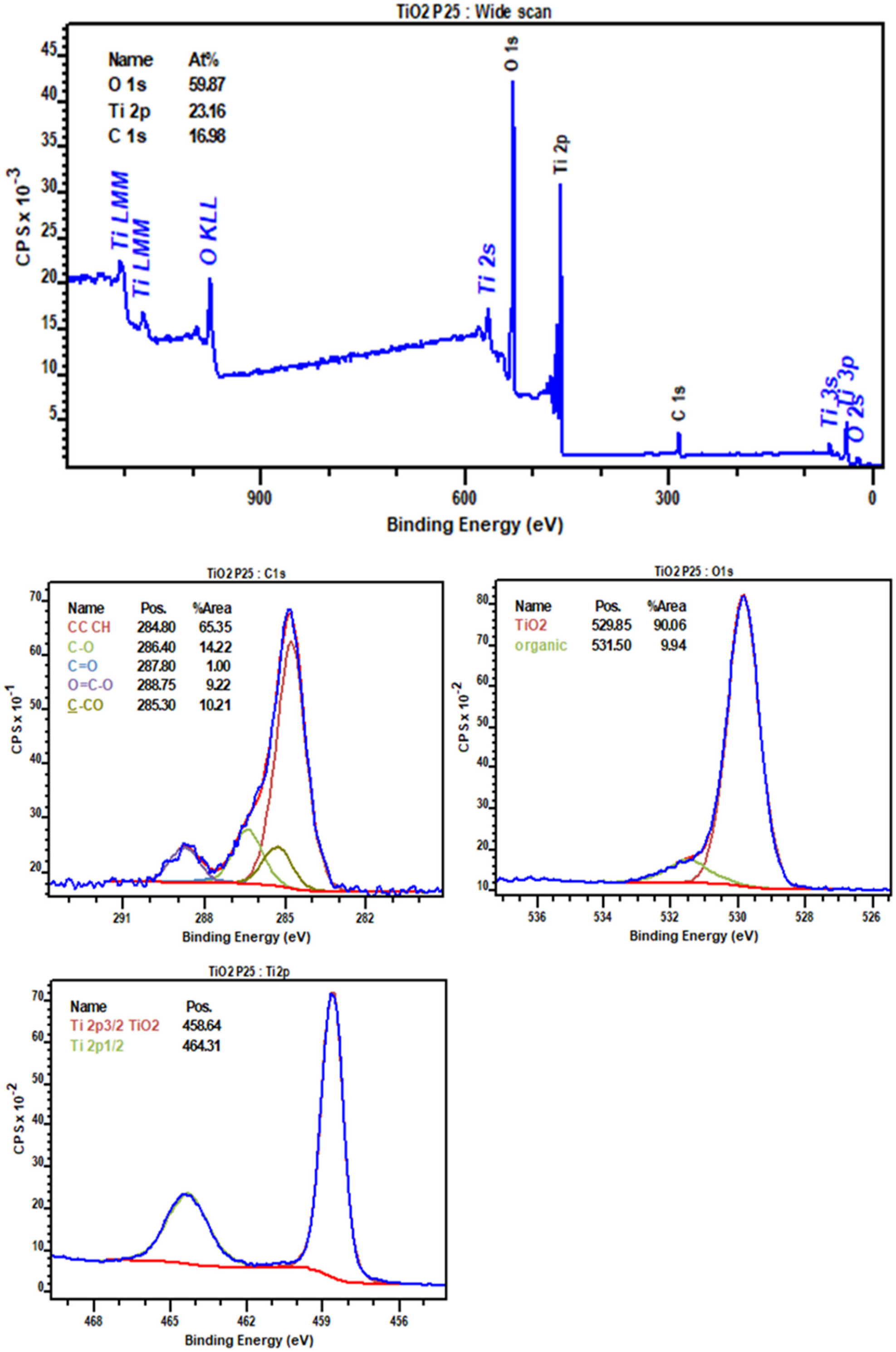
TiO2 P25 : Wide scan
Name At%
O 1s 59.87
Ti 2p 23.16
C 1s 16.98
Ti LMM
Ti LMM
O KLL
Ti 2s
O 1s
Ti 2p
C 1s
Ti 3s
Ti 3p
O 2s
CPS x 10-3
Binding Energy (eV)
TiO2 P25 : C1s
Name Pos. %Area
CC CH 284.80 65.35
C-O 286.40 14.22
C=O 287.80 1.00
O=C-O 288.75 9.22
C-CO 285.30 10.21
CPS x 10-1
Binding Energy (eV)
TiO2 P25 : O1s
Name Pos. %Area
TiO2 529.85 90.06
organic 531.50 9.94
CPS x 10-2
Binding Energy (eV)
TiO2 P25 : Ti2p
Name Pos.
Ti 2p3/2 TiO2 458.64
Ti 2p1/2 464.31
CPS x 10-2
Binding Energy (eV)

(**a**)

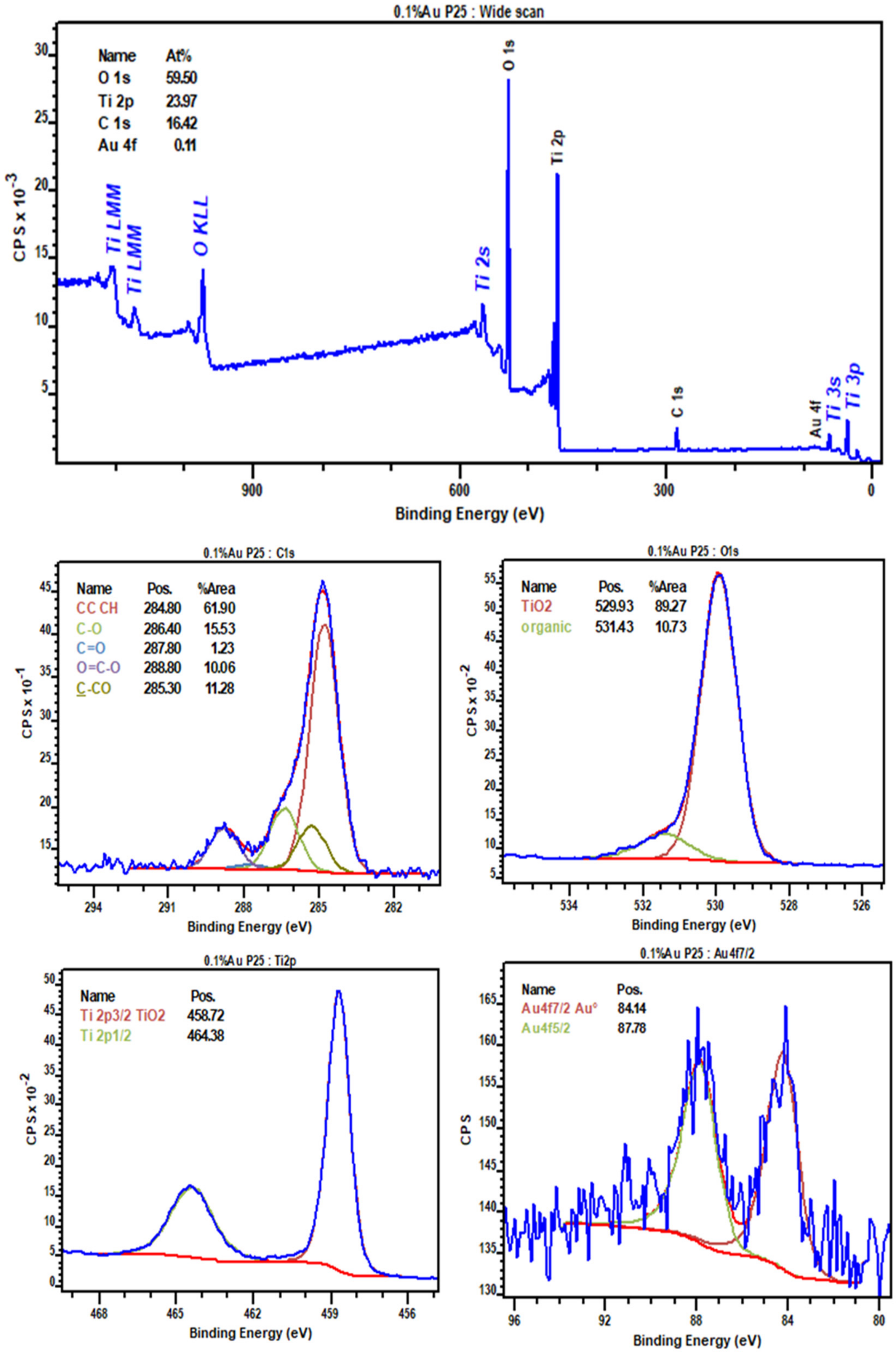
0.1%Au P25 : Wide scan
Name At%
O 1s 59.50
Ti 2p 23.97
C 1s 16.42
Au 4f 0.11
CPS x 10-3
Ti LMM
Ti LMM
O KLL
Ti 2s
O 1s
Ti 2p
C 1s
Au 4f
Ti 3s
Ti 3p
Binding Energy (eV)
0.1%Au P25 : C1s
Name Pos. %Area
CC CH 284.80 61.90
C-O 286.40 15.53
C=O 287.80 1.23
O=C-O 288.80 10.06
C-CO 285.30 11.28
CPS x 10-1
Binding Energy (eV)
0.1%Au P25 : O1s
Name Pos. %Area
TiO2 529.93 89.27
organic 531.43 10.73
CPS x 10-2
Binding Energy (eV)
0.1%Au P25 : Ti2p
Name Pos.
Ti 2p3/2 TiO2 458.72
Ti 2p1/2 464.38
CPS x 10-2
Binding Energy (eV)
0.1%Au P25 : Au4f7/2
Name Pos.
Au4f7/2 Au° 84.14
Au4f5/2 87.78
CPS
Binding Energy (eV)

(**b**)

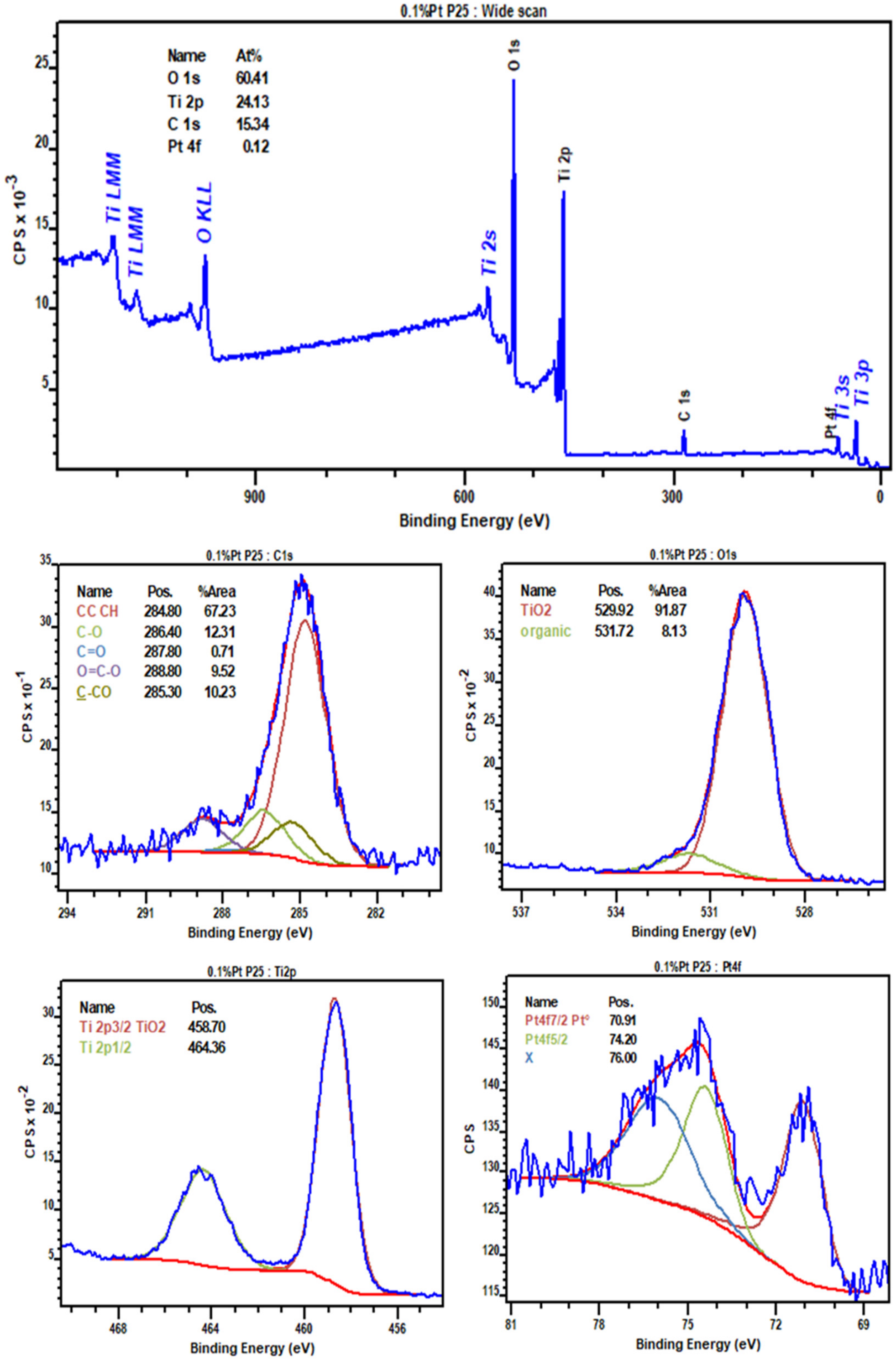
0.1%Pt P25 : Wide scan
Name At%
O 1s 60.41
Ti 2p 24.13
C 1s 15.34
Pt 4f 0.12
Ti LMM
Ti LMM
O KLL
Ti 2s
O 1s
Ti 2p
C 1s
Pt 4f
Ti 3s
Ti 3p
CPS x 10-3
Binding Energy (eV)
0.1%Pt P25 : C1s
Name Pos. %Area
CC CH 284.80 67.23
C-O 286.40 12.31
C=O 287.80 0.71
O=C-O 288.80 9.52
C-CO 285.30 10.23
CPS x 10-1
Binding Energy (eV)
0.1%Pt P25 : O1s
Name Pos. %Area
TiO2 529.92 91.87
organic 531.72 8.13
CPS x 10-2
Binding Energy (eV)
0.1%Pt P25 : Ti2p
Name Pos.
Ti 2p3/2 TiO2 458.70
Ti 2p1/2 464.36
CPS x 10-2
Binding Energy (eV)
0.1%Pt P25 : Pt4f
Name Pos.
Pt4f7/2 Pt° 70.91
Pt4f5/2 74.20
X 76.00
CPS
Binding Energy (eV)

(**c**)

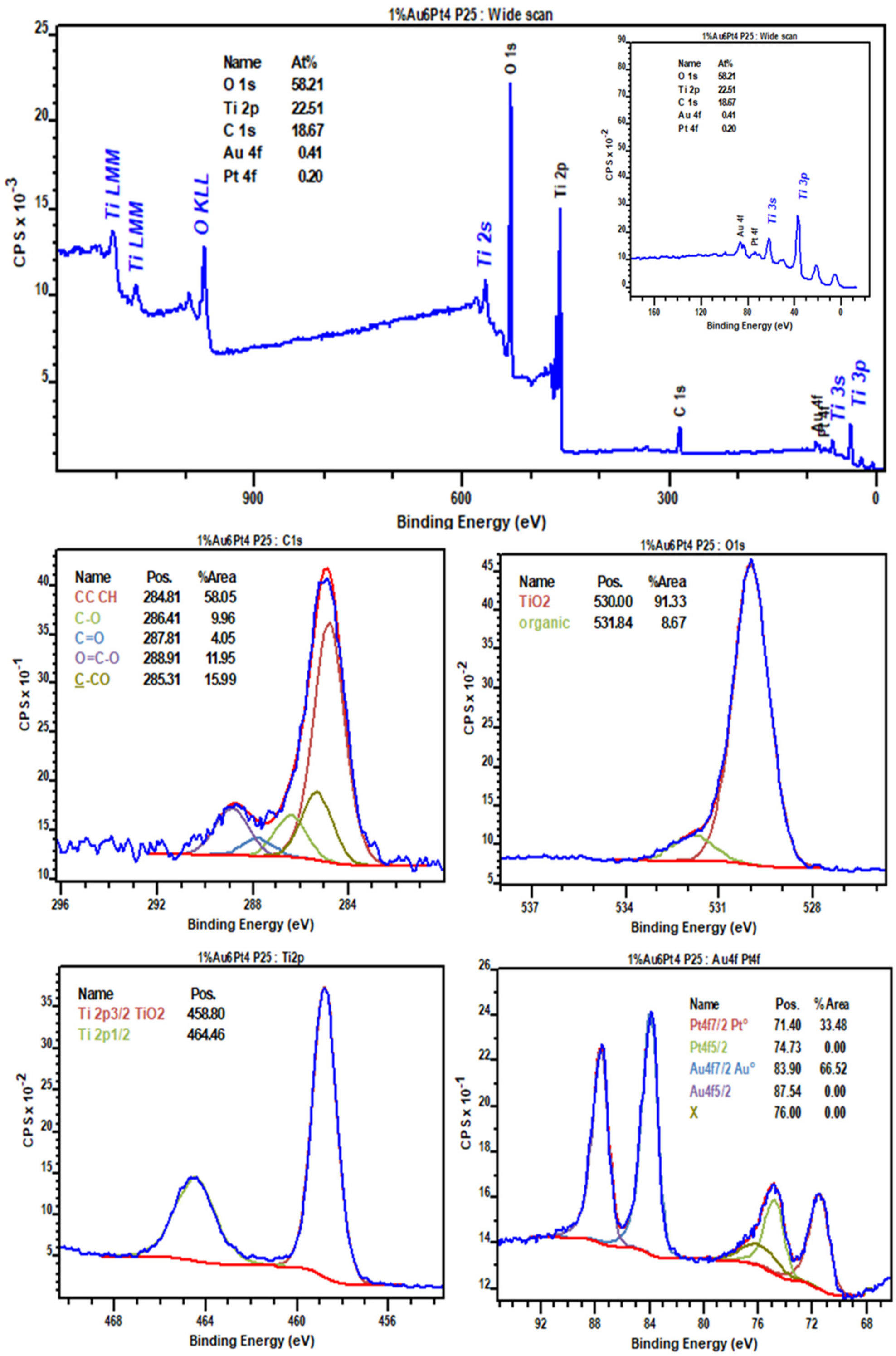
1%Au6Pt4 P25 : Wide scan
Name At%
O 1s 58.21
Ti 2p 22.51
C 1s 18.67
Au 4f 0.41
Pt 4f 0.20
Ti LMM
Ti LMM
O KLL
Ti 2s
O 1s
Ti 2p
C 1s
Au 4f
Pt 4f
Ti 3s
Ti 3p
CPS x 10-3
Binding Energy (eV)
1%Au6Pt4 P25 : C1s
Name Pos. %Area
CC CH 284.81 58.05
C-O 286.41 9.96
C=O 287.81 4.05
O=C-O 288.91 11.95
C-CO 285.31 15.99
CPS x 10-1
Binding Energy (eV)
1%Au6Pt4 P25 : O1s
Name Pos. %Area
TiO2 530.00 91.33
organic 531.84 8.67
CPS x 10-2
Binding Energy (eV)
1%Au6Pt4 P25 : Ti2p
Name Pos.
Ti 2p3/2 TiO2 458.80
Ti 2p1/2 464.46
CPS x 10-2
Binding Energy (eV)
1%Au6Pt4 P25 : Au4f Pt4f
Name Pos. %Area
Pt4f7/2 Pt° 71.40 33.48
Pt4f5/2 74.73 0.00
Au4f7/2 Au° 83.90 66.52
Au4f5/2 87.54 0.00
X 76.00 0.00
CPS x 10-1
Binding Energy (eV)

(**d**)

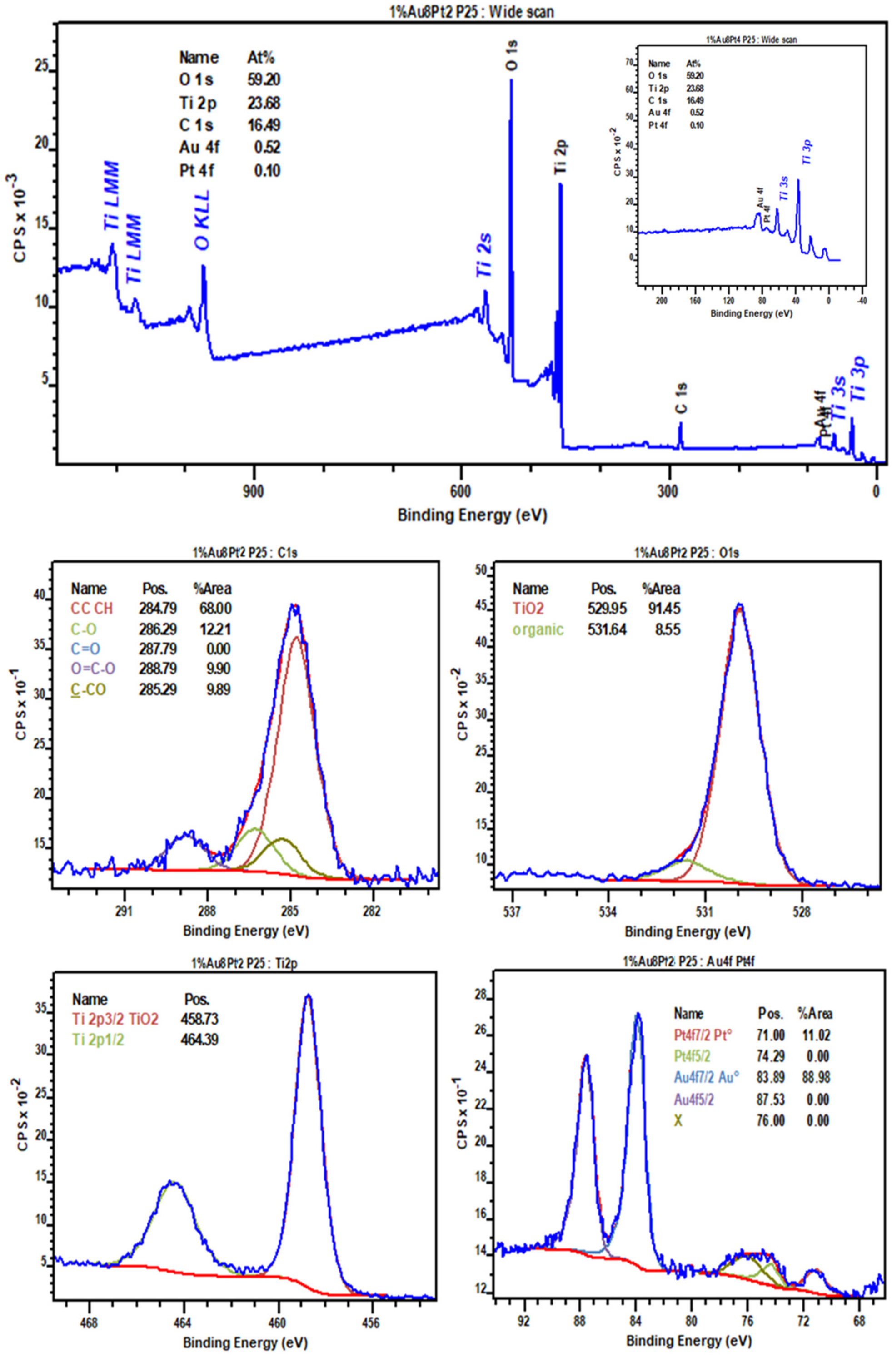

(**e**)

Figure S4. Full XPS spectra: (**a**) P25, (**b**) 0.1%molAu, (**c**) 0.1%molPt, (**d**) 1%wt(Au_6Pt_4) and (**e**) 1%wt(Au_8Pt_2).

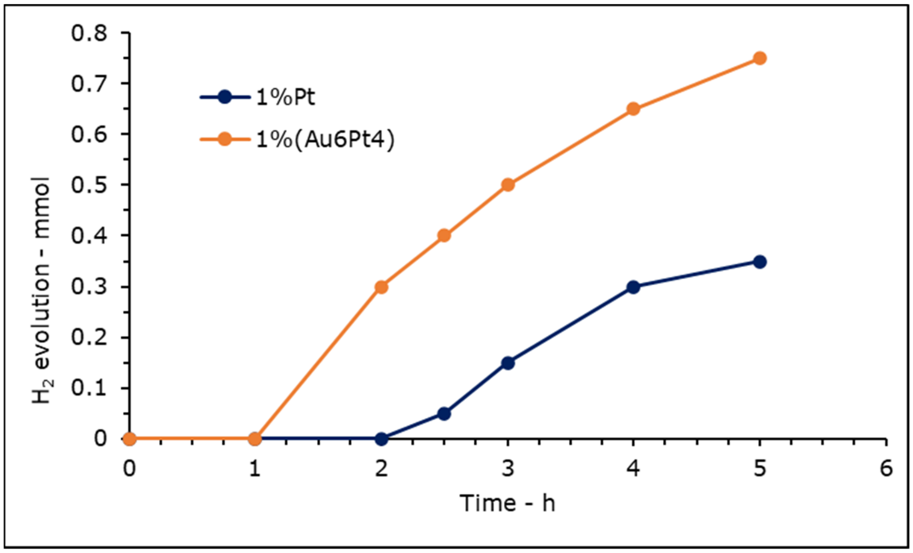

Figure S5. Hydrogen evolution of photo-reforming process for the best mono and bi-metallic photocatalysts.
